# Allelic and genotypic frequencies of SNP related to beef and carcass quality in Romosinuano cattle in Mexico

**DOI:** 10.1007/s11250-023-03643-y

**Published:** 2023-06-07

**Authors:** Ricardo Martínez Rocha, Rodolfo Ramírez-Valverde, Rafael Núñez-Domínguez, José G. García-Muñiz, Gaspar M. Parra-Bracamonte, Joel Domínguez-Viveros

**Affiliations:** 1 Facultad de Estudios Superiores Cuautitlán, Universidad Nacional Autónoma de México, Carr. Cuautitlán-Teoloyucan Km 2.5, Cuautitlán, Estado de México México; 2 Universidad Autónoma Chapingo, Posgrado en Producción Animal, Km 38.5 Carretera México-Texcoco, Chapingo, Estado de México México; 3 Centro de Biotecnología Genómica, Instituto Politécnico Nacional, Boulevard del Maestro SN. Esq. Elías Piña, Col. Narciso Mendoza, Reynosa, Tamaulipas México; 4 Facultad de Zootecnia y Ecología, Universidad Autónoma de Chihuahua, Km 1, Periférico Francisco R Almada, Chihuahua Chihuahua, México

**Keywords:** Creole cattle, Tropically adapted breed, Meat quality

## Abstract

Romosinuano is a tropically adapted *Bos taurus* breed, and some Mexican breeders aim to improve it genetically. The aim was to estimate allelic and genotypic frequencies for SNPs associated with meat quality in a Mexican Romosinuano population. Four hundred ninety-six animals were genotyped using the Axiom©BovMDv3 array. Only SNPs related to meat quality in this array were studied in this analysis. The Calpain, Calpastatin, and Melanocortin-4 receptor alleles were considered. Allelic and genotypic frequencies and Hardy-Weinberg equilibrium were estimated with the PLINK software. Alleles associated with meat tenderness and higher marbling scores were found in the Romosinuano cattle population. CAPN1_4751 was not found in Hardy-Weinberg equilibrium. The rest of the markers could not be affected by selection and inbreeding. Romosinuano cattle in Mexico have similar genotypic frequencies in markers related to meat quality to *Bos taurus* breeds known for their meat tenderness. Breeders can choose a marker-assisted selection to improve meat quality characteristics.

## Introduction

Zebu cattle are widely utilized as a genetic resource for beef production in the tropics of Mexico due to their adaptation to high temperatures and humidity (Segura-Correa et al. 2017). Most *Bos taurus* breeds predominantly found in countries with temperate weather have a high-production potential but poor adaptation to the harsh tropical environment (Roschinsky et al. 2015). Nevertheless, it is well known that meat obtained from *Bos indicus* cattle breeds is less tender than meat from *Bos taurus* cattle. Beef’s palatability and tenderness are important characteristics that influence consumer demand (Crouse et al. 1989). The cattle must be crossbreed (*Bos taurus* × *Bos indicus*) or tropically adapted *Bos taurus* breed to produce more tender beef in Mexico’s tropical region.

Creole cattle breeds (creole refers to the cattle descendant of bovines introduced to the New World from the Andalucia region of southern Spain) showed no differences in tenderness of meat compared with Angus and Hereford (Anderson et al. 2015). Some Creole breeds have been developed in adverse climate conditions, e.g., the tropically adapted Romosinuano of Colombia. Several Mexican breeders have used Romosinuano cattle due to their characteristics of adaptation to tropical climates (Núñez-Domínguez et al. 2020).

Nevertheless, they do not have selection criteria for improving meat quality yet. Marker-assisted genomic selection could be suitable for achieving genetic progress in meat quality. Thus, this study aimed to estimate allelic and genotypic frequencies for SNPs associated with meat quality in a Mexican Romosinuano population.

## Material and methods

Samples of hair follicles were collected from 496 Romosinuano animals registered in the *Asociación Mexicana de Criadores de Ganado Romosinuano y Criollo Lechero Tropical, A. C.* (AMCROLET). The sampled animals were born between 2003 and 2019, including 49 males and 447 females, from four farms located in Mexico’s tropical areas. In general, genetic material is continuously exchanged among these farms, providing most of the sires, semen, and females to other breeders (Núñez-Dominguez et al. 2020). Animals were genotyped using 63,791 SNPs from the Axiom©BovMDv3 array from Affymetrix (Santa Clara, CA, USA). Markers were identified using the genomic information available for *Bos taurus* at National Center for Biotechnology Information (2021). Only markers in the SNP chip used could be considered.

The markers in the SNP chip used were Calpain (CAPN1_316 CAPN1_530 CAPN1_4751), Calpastatin (CAST_282, CAST_2870, CAST_2959), Myostatin (MYO_F94L, MYO_S105C, MYO_Q204X, MYO_E226X, MYO_821del11, MYO_E291X, MYO_C313Y), thyroglobulin (TG), diacylglycerol O-acyltransferase 1 (DGAT1), and melanocortin-4 receptor (MC4R). Allele and genotypic frequencies were calculated with PLINK v1.9 software (Purcell et al. 2007). Hardy-Weinberg equilibrium test was also estimated for each desired allele (Ho: F_OB_ = F_EXP_). The maximum per SNP missing of 0.1 was considered the inclusion threshold.

## Results

The markers for DGAT1 and TG had a missing SNP genotype ratio smaller than 0.9. When related to target traits, missing SNP genotypes would confound data analyses. SNPs at the myostatin gene were not presented as a polymorphic form in Romosinuano cattle. Genotypic and allelic frequencies, the position of each marker, and the Hardy-Weinberg equilibrium (HWE) test are presented in Table 1.

**Table 1:** Genotypic and allelic frequencies of markers previously associated with meat quality present in the Mexican Romosinuano population

Gene	Marker	Chr	Position					HWE (*p*-value)
				Genotype	Freq	Allele	Freq	
Calpain	CAPN1_316	29	44069063	CC	0.02	C	0.16	0.13
				CG	0.30			
				GG	0.68	G	0.84	
	CAPN1_4751	29	44087629	CC	0.21	C	0.495	< 0.01
				CT	0.57			
				TT	0.22	T	0.505	
	CAPN1_530	29	44085642	GG	0.03	G	0.190	0.23
				GC	0.32			
				CC	0.66	C	0.810	
Calpastatin	CAST_282	7	98533961	CC	0.17	C	0.435	0.10
				CG	0.53			
				GG	0.30	G	0.565	
	CAST_2870	7	98579574	GG	0.18	G	0.445	0.08
				GA	0.53			
				AA	0.29	A	0.555	
	CAST_2959	7	98579663	AA	0.11	A	0.35	0.33
				AG	0.48			
				GG	0.41	G	0.65	
MC4R	MC4R	24	59670860	CC	0.23	C	0.49	0.37
				CG	0.52			
				GG	0.25	G	0.51	

Abbreviations: *Chr* chromosome, *Freq* frequency, *HWE* Hardy-Weinberg equilibrium test (if *p*-value < 0.05, reject Ho: F_Ob_ = F_Exp_)

## Discussion

SNP genotyping assays normally give rise to a certain percentage of no-calls. Only one marker did not present Hardy-Weinberg equilibrium in the population. This method assumes that genotype frequencies should comply with HWE proportions using the chi-squared test in a large and random mating population. Many factors can cause deviation from these proportions: a genotyping error, purifying selection, copy number variation, inbreeding, or population substructure (Chen et al. 2017; Ocampo et al. 2021). Although the Mexican Romosinuano had been forced to be a genetically closed population due to sanitary regulations that would not allow the import of genetic material directly from Colombia, the selection realized by breeders and their reduced genetic diversity (Núñez Dominguez et al. 2020), most of these markers were in Hardy-Weinberg equilibrium.

The Calpain1 protease breaks down myofibrillar (muscle fiber) proteins post-mortem. This gene has three markers (CAPN1 316, CAPN1 4751, and CAPN1 530) associated with meat tenderness. These are not causative mutations, but they are predictors of tenderness in multiple cattle breeds; the markers each explain from 0.4 to 2% of the phenotypic variation in tenderness, with the amount explained varying across breeds (McClure and McClure 2016).

CAPN1_316 (c.947G>C) and CAPN1_530 (c.1588G>A) showed a low frequency of the favorable homozygote. It has been reported that genotype CC in CAPN1_316 is associated with higher marbling scores than the other genotypes (Li et al. 2013). Bonilla et al. (2010) also reported low proportions of the favorable homozygote in Mexican crossbreed cattle. Three Bolivian creole cattle (Yacumeño, Saavedreño, and Caqueteño) showed a low frequency of favorable homozygotes (Pereira et al. 2015). Cuetia et al. (2012) also found a low frequency of CAPN1_316 with SSCP markers. The trait allele of CAPN1_4751 was found in 49 % of the studied cattle. This marker was the only one that was not in H-W equilibrium (*p* < 0.05). In other studies, cattle populations also presented similar frequencies between trait allele and normal allele (Bonilla et al. 2010; Pereira et al. 2015; Parra-Bracamonte et al. 2009). CAPN1_530 also showed a low frequency of the trait allele. GG genotype at marker 530 increased meat tenderness compared with AG and AA genotypes (Page et al. 2002). C/G haplotype in CAPN1 316 and 530 has been associated with meat tenderness in cattle (Coria et al. 2018).

CAST is a naturally occurring protein that inhibits the normal tenderization of meat as it ages post-mortem (Schenkel et al. 2006). CAST inhibits calpain activity and, therefore, normalizes post-mortem proteolysis; if there is an increase in post-mortem CAST activity, meat tenderness is reduced (McClure and McClure 2016). Three variants of the CAST gene (CAST_282, CAST_2870, CAST 2959) have been found to affect beef tenderness. In Romosinuano cattle, similar frequencies in CAST_282 and CAST_2870 were found. The allele frequencies of CAST_282 were consistent with previous studies on Angus and Piedmontese (Ribeca et al. 2013; Ruban et al. 2017). In contrast, some Italian cattle breeds had higher allele frequencies (Lisa and Stasio 2009).

The melanocortin-4 receptor (MC4R) is associated with energy homeostasis. It was also considered a positional candidate gene for final body weight, backfat thickness, and marbling score (Liu et al. 2010; Mazzucco et al. 2016). MC4R allele (rs108968214 C/G) was in polymorphic form in Romosinuano cattle. Mazzuco et al. (2016) reported low frequencies (0.0 to 0.07) of the GG genotype in Angus, Hereford, and crossbreed steers. GG genotype was found in 22.8 % of the Romosinuano cattle. Liu et al. (2010) reported that cattle with GG and CG genotypes had higher marbling scores than CC.

Romosinuano cattle in Mexico have similar genotypic frequencies in markers related to meat quality to *Bos taurus* breeds known for their meat tenderness. The majority of markers was in Hardy-Weinberg equilibrium. Breeders can choose a marker-assisted selection as a complementary tool for conventional genetic selection or genomic selection to improve meat quality characteristics.

## Data Availability

The dataset generated by the current study is not publicly available since it belongs to AMCROLET and is treated as confidential information.
